# Characterization of the C6D7-RBD Human Monoclonal Antibody Specific to the SARS-CoV-2 S Protein Receptor-Binding Domain

**DOI:** 10.32607/actanaturae.11849

**Published:** 2023

**Authors:** Ya. O. Romanenko, M. V. Silkina, A. S. Kartseva, M. A. Marin, M. A. Shkuratova, M. A. Makarova, A. K. Ryabko, D. A. Konyshkova, N. A. Zeninskaya, A. E. Khlyntseva, I. G. Shemyakin, V. V. Firstova

**Affiliations:** Federal Budget Institution of Science State Research Center for Applied Microbiology and Biotechnology, Obolensk, Moscow region, 142279 Russian Federation

**Keywords:** COVID-19, SARS-CoV-2, receptor-binding domain, human monoclonal antibody, virus-neutralizing activity

## Abstract

The new coronavirus infection COVID-19 is an acute viral disease that affects primarily the upper respiratory tract. The etiological agent of COVID-19 is the SARS-CoV-2 RNA virus (Coronaviridae family, Betacoronavirus genus, Sarbecovirus subgenus). We have developed a high-affinity human monoclonal antibody, called C6D7-RBD, which is specific to the S protein receptor-binding domain (RBD) from the SARS-CoV-2 Wuhan-Hu-1 strain and exhibits virus-neutralizing activity in a test with recombinant antigens: angiotensin-converting enzyme 2 (ACE2) and RBD.

## INTRODUCTION

h e n e w c o r o n av i r u s i n f e c t i o n C OV I D - 1 9 (COronaVIrus Disease 2019), which is caused by the severe acute respiratory syndrome-related coronavirus 2 (SARS-CoV-2), was first detected in Wuhan, the capital of China’s Hubei province, at the end of 2019. Despite all efforts to contain the disease in China, the virus spread across the world, and soon the World Health Organization (WHO) had declared a COVID-19 pandemic [1]. To date, the ongoing pandemic continues, claiming many more lives. Many effective vaccines have been developed around the world over that short period of time. However, there remains a need for agents that can be used for passive immunotherapy of moderate and severe cases of the disease; in particular, human monoclonal antibody (hMAb)-based medications.

The SARS-CoV-2 virus genome encodes four structural proteins: the surface spike (S) glycoprotein, the membrane (M) protein, nucleocapsid (N) protein, and the envelope (E) protein. The S protein mediates viral attachment, fusion, and entry into the host cell. The transmembrane serine protease 2 (TMPRSS2) cleaves the S protein into two subunits, S1 and S2 [2, 3]. The receptor interaction between the virus and the host cell is mediated by RBD that is located in the S1 subunit. Next, the S2 subunit promotes fusion of the viral membrane with that of the host cell [4]. Therefore, RBD is the main target in developing hMAbs capable of neutralizing the virus [5].

As the disease has spread and mortality rates have increased, researchers around the world have scrambled to come up with innovative agents with high clinical efficacy and safety.

In October 2020, the Ministry of Health of the Russian Federation approved the use of plasma from convalescent donors (surviving patients diagnosed with COVID-19) for the treatment of patients with a new severe coronavirus infection because of the lack of drugs for a specific treatment. According to their guidelines, the age range of potential donors should be 18 to 55 years, the body weight should exceed 55 kg, and the blood plasma should be collected no earlier than 14 days after resolving clinical symptoms and receiving a double negative oropharyngeal swab for SARS-CoV-2 RNA performed in a 24-hour or more interval. The blood plasma should exhibit virus-neutralizing activity at a 1 : 160 dilution; the total protein concentration in the blood should not be less than 65 g/L [6].

In the Russian Federation, there is no experience of the use of monoclonal antibody-based drugs on severely-ill patients, but there is one patented hMAb that exhibits neutralizing activity and selectively interacts with a SARS-CoV-2 S protein RBD fragment [7].

The development of hMAbs specific to the SARS-CoV-2 S protein RBD is promising. This study characterizes the developed hMAb, which may be used for the treatment of COVID-19.

## EXPERIMENTAL

For this study, we chose a blood donor who had recovered from the new coronavirus infection COVID-19 and was vaccinated with the Sputnik Light vaccine (manufactured by the Gamaleya National Research Center for Epidemiology and Microbiology, the Russian Academy of Medical Sciences) 6 months after recovery. The donor provided written informed consent to participate in the study. On day 7 after vaccination, peripheral blood was collected and the B lymphocyte fraction was isolated using a RossetteSep™ Human B Cell Enrichment Cocktail kit (Stemcell technologies, Canada), in accordance with the manufacturer’s instructions. The isolated B lymphocytes were electrofused with the K6H6/B5 myeloma cell line (ATCC® CRL1823™) using an ECM 2001 device (BTX Harvard Apparatus, USA), according to a previously published procedure [8].

The produced hybridomas were cultured at a temperature of 37°C in a humid atmosphere with 5% CO_2_. The culture medium was replaced once every three days. After the hybrid culture reached the monolayer, an enzyme-linked immunosorbent assay (ELISA) was performed to identify hybridomas producing SARS-CoV-2 S protein RBD specific to mAbs.

**Enzyme-linked immunosorbent assay**

The culture fluid from the wells with grown hybrid cells was tested using ELISA for specific interactions with a recombinant RBD from the SARS-CoV-2 Wuhan-Hu-1 strain (his-sars2-rbd, Invivogen). For this, 100 μL of a solution of the recombinant RBD (his-sars2-rbd, Invivogen) at a concentration of 1 μg/mL per well in PBS was added to the wells of a 96-well polystyrene plate and incubated at a temperature of 37°C on an orbital plate shaker (Elmi, Latvia) at 370 rpm for 2 h. Then, each well of the plate was washed three times with 200 μL of PBS containing 0.05% Tween-20 (PBS–TW). Later, the free binding sites on the wells were blocked with milk with a fat content of no more than 0.5% (200 μL per well) and incubated at 37°C h under the same conditions for 1h. After incubation, the wells were washed with PBS-TW three times. Next, 100 µL of the culture liquid was added to the plate and the plate was incubated and washed under the same conditions. The wells containing PBS alone were used as a negative control; the donor’s pretested blood serum with a high titer of anti-RBD antibodies at a 1 : 25 dilution was used as a positive control. After incubation, the wells were washed with PBS-TW three times. Then, anti-human IgG (whole molecule) rabbit antibodies conjugated with horseradish peroxidase (Sigma, USA) at a 1 : 20 000 dilution were added to the wells. A plate was incubated under the same conditions for 40 min. At the end of the incubation, the plate was washed 6 times and 100 μL of a developing solution containing 3,3’,5,5’-tetramethylbenzidine (TMB) was added to the wells of the plate. The reaction was assessed based on the development of a blue color. The staining intensity was measured on a microplate spectrophotometer (Bio-Rad xMark) at a wavelength of 655 nm.

Hybrid cell colonies displaying high absorbance (3-fold higher than the negative control value) in ELISA were cloned and scaled in Corning® T-25 and T-75 culture flasks. Then, the hybrid culture was cultivated in 1.6 L Optimum Growth™ flasks (Thomson Instrument Company, USA) to produce hMAbs.

**Affinity chromatography**

To produce the C6D7-RBD hMAb, the culture liquid used for culturing hybridomas was purified by affinity chromatography on a Protein G-sepharose column (HiTrap™ Protein G, Sweden) using an ÄKTA Start system (GE Healthcare, USA). The isolated IgG was transferred into PBS and purified by gel filtration on a Superdex 200 10/300 GL sorbent (GE Healthcare, USA). The purity of the resulting immunoglobulin fraction was evaluated by Laemmli SDS electrophoresis in 10% polyacrylamide gel (PAG) under reducing and non-reducing conditions. Gels were stained with Coomassie Brilliant Blue R-250.

**Immunoblot analysis**

The immunological specificity of the purified C6D7- RBD hMAb was assessed by immunoblotting. For this, the recombinant RBD (1 μg per lane) was separated by PAG electrophoresis under reducing conditions. Further, the protein was horizontally transferred from the gel to a Hybond-C Extra nitrocellulose membrane (GE Healthcare) using a standard procedure. After transfer, the membrane was immersed in skimmed milk (less than 0.5% fat) to block the free binding sites on the nitrocellulose and incubated at a temperature of 37°C with shaking for 1 h. After incubation, the membrane was washed with PBS-TW three times and immersed in a 10 µg/mL C6D7-RBD hMAb solution in PBS. The membrane was incubated and washed under the same conditions. The hMAb on the membrane was detected using anti-human IgG goat antibodies conjugated with horseradish peroxidase (Sigma) at a 1 : 10 000 dilution in PBS. The membrane was incubated under the same conditions for 40 min. After incubation, the membrane was washed with PBS-TW 6 times and developed with a 1% diaminobenzidine solution in PBS supplemented with nickel and cobalt chlorides and 33% hydrogen peroxide (1 μL per 1 mL of a developing solution).

**Class assignment of C6D7-RBD hMAb**

The subclass and isotype of the purified C6D7-RBD hMAb were assessed using an immunochromatographic rapid test (Iso-Gold™ Rapid Human Antibody Isotyping Kit, Canada). All reagents were brought to room temperature before the testing. A C6D7-RBD hMAb test sample was diluted 100-fold with 200 μL of sample dilution buffer (Part Number SDB-004). Then, a test strip was immersed in a tube with the test sample. The result was analyzed after 5–10 min.

**Determination of the equilibrium dissociation constant for C6D7-RBD hMAb and the target RBD protein**

The equilibrium dissociation constant for C6D7-RBD hMAb and the target RBD protein was determined using surface plasmon resonance (SPR) spectroscopy on a BIAcore X-100 instrument (Biacore, Sweden). The experiment was performed on a CM5 sensor chip in HBS-EP buffer (10 mM Hepes, 150 mM NaCl, 3 mM EDTA, and 0.005% P20 surfactant, pH 7.4). Anti-histidine antibodies were conjugated to the sensor chip using a His Capture Kit Type 2 and an Amine Coupling Kit (Cytiva, Sweden), according to the manufacturer’s instructions. The recombinant RBD (10 μg/mL) was applied to the prepared chip at a rate of 30 μL/min for 3 min. After a 10-minute stabilization period, the antibody (concentrations of 6.25 to 100 nM) was injected at a constant flow rate of 40 μL/min for 3 min. Dissociation was monitored for 90 min. Next, the chip was regenerated with 10 mM glycine pH 1.7 at a flow rate of 50 µL/min for 30 s. Sensorograms were normalized by subtracting baseline RU values from the reference flow cell (no hMAb capture) and analyzed by fitting the data to a 1 : 1 Langmuir binding model using the Biacore T200 Evaluation software.

**C6D7-RBD hMAb-mediated inhibition of the ACE-2–RBD interaction**

The virus-neutralizing activity of C6D7-RBD hMAb was evaluated using competitive ELISA. For this purpose, the recombinant RBD protein was immobilized on the wells of a 96-well polystyrene plate at a concentration of 1 μg/mL according to the above-described procedure. Then, the C6D7-RBD hMAb was added to the wells at concentrations of 10 to 0.078125 μg/mL in a twofold serial dilution step. The plate was incubated at 37°C for 1 h. After incubation, the wells were washed with PBS-TW three times. Then, recombinant human ACE-2 (fc-hace2, Invivogen) conjugated with horseradish peroxidase was added to the wells using a LYNX Rapid HRP Antibody Conjugation kit (Bio-Rad). The plate was incubated and washed using the same conditions. Then, 100 μL of a TMB developing solution was added to the wells. The reaction was evaluated based on the development of a blue color. The staining intensity was measured on a microplate spectrophotometer (Bio-Rad xMark) at a wavelength of 655 nm. The mean background optical density (control of the wells without RBD immobilization, which is equivalent to the RBD completely blocked by antibodies) was considered as 100% neutralizing activity. The mean optical density of the control wells where ACE-2–HRP interacted with RBD in the absence of antibodies was considered as a lack of neutralizing activity (0%). The control values were used to generate a linear function in order to quantify absorbance in the experimental wells with varying antibody amounts as the percentage of neutralizing activity.

## RESULTS

Electrofusion of plasmablasts with K6H6/B5 partner cells resulted in 5 hybridomas. Of these, one hybridoma, called C6D7-RBD, was selected based on the results of a screening for the specificity of the anti-RBD antibodies synthesized by the hybridomas. Subsequent scaling of the hybridoma culture provided a large amount of supernatant with antibodies. A pure immunoglobulin fraction was produced using chromatographic purification techniques.

**Chromatographic purification of C6D7-RBD hMAb**

**Fig. 1:**
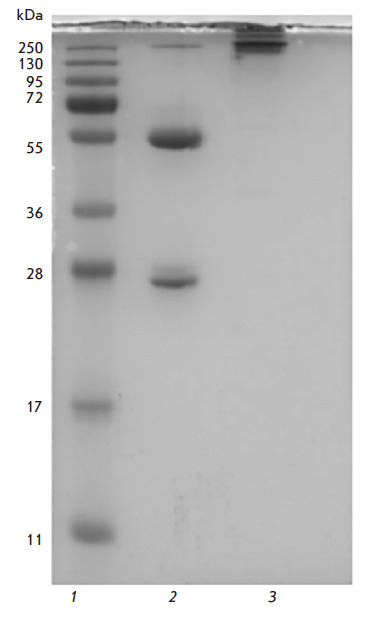
Electrophoregram of C6D7-RBD hMAb. *1 *– PageRuler™ SM1811 molecular weight markers (Fermentas, USA). *2 *– C6D7-RBD hMAb sample, reducing conditions. *3 *– C6D7-RBD hMAb sample, non-reducing conditions

The purity of the immunoglobulin fraction produced by affinity chromatography and subsequent gel filtration was assessed by Laemmli SDS-electrophoresis in 10% PAG under reducing and non-reducing conditions (Fig. 1). The position of the purified C6D7-RBD hMAb on the electropherogram corresponds to its molecular weight. Based on densitometry data (GE Typhoon FLA 9500, Sweden), the purity of C6D7-RBD hMAb amounted to at least 95%.

**Immunological specificity of C6D7-RBD hMAb to the SARS-CoV-2 S protein receptor-binding domain**

**Fig. 2:**
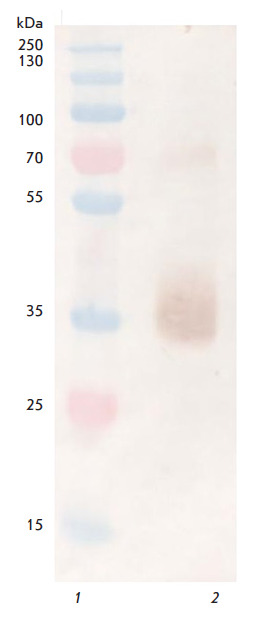
C6D7-RBD hMAb specificity to the recombinant RBD protein. *1 *– PageRuler™ SM1811 molecular weight markers (Fermentas, USA). *2 *– Recombinant RBD detected with C6D7-RBD hMAb

The specificity of C6D7-RBD hMAb to the recombinant RBD protein was confirmed by immunoblotting. The C6D7-RBD hMAb was shown to exhibit specific activity against the recombinant RBD protein (Fig. 2).

**Immunochromatographic test results**

**Fig. 3:**
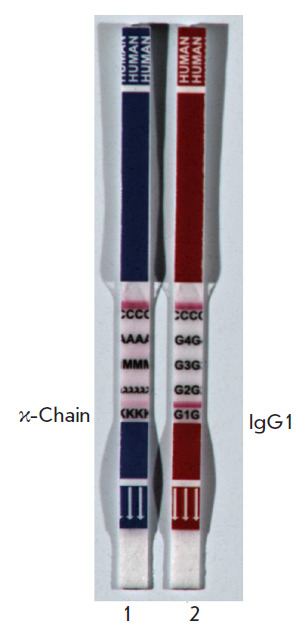
Class assignment of C6D7-RBD hMAb. *1 *– Strip for determining the light chain type and immunoglobulin class. *2 *– Strip for determining the IgG subclass

According to the immunochromatographic test data, the C6D7-RBD hMAb is the G1 isotype of class G immunoglobulins and contains the κ-chain (Fig. 3).

**Determination of the equilibrium dissociation constant for the C6D7-RBD hMAb and the target RBD protein (Wuhan)**

**Fig. 4:**
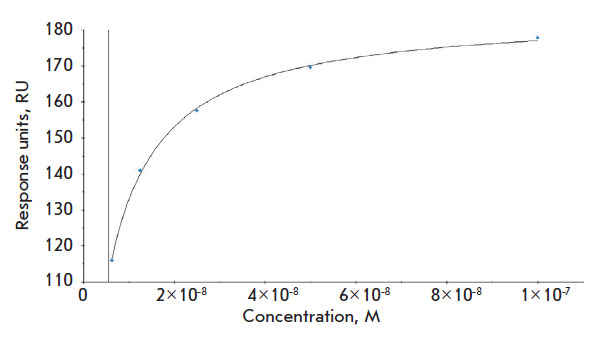
Determining *K*_D_ for C6D7-RBD hMAb and the target RBD protein

The parameters of the affinity interaction between the C6D7-RBD hMAb and the recombinant RBD protein were evaluated using surface plasmon resonance (SPR) spectroscopy. The equilibrium dissociation constant (KD) for the C6D7-RBD hMAb was KD = 5.525 × 10^–9^ M (Fig. 4).

**RBD–ACE2-neutralizing activity of the C6D7-RBD hMAb**

**Fig. 5:**
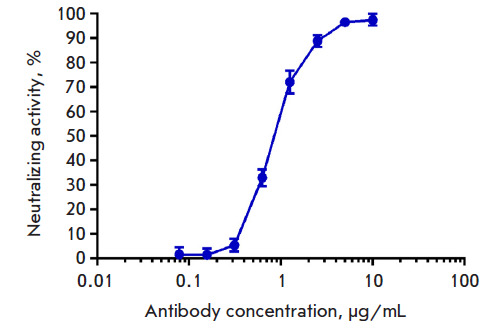
RBD–ACE2-neutralizing activity of C6D7-RBD hMAb

Figure 5 shows a dose-dependent increase in the neutralizing activity of C6D7-RBD hMAb. Almost complete (97%) inhibition of the ACE-2–RBD (Wuhan) interaction was observed at a maximum C6D7-RBD hMAb concentration of 10 μg/mL. Addition of the C6D7-RBD hMAb to a concentration of 0.625 µg/mL blunted the ACE-2–RBD (Wuhan) interaction by 36%; there was almost no neutralizing activity (2%) at the minimum C6D7-RBD hMAb concentration (0.078125 μg/mL).

## DISCUSSION

Production of hMAb with naturally paired heavy and light chains which is specific to a chosen target and exhibits virus-neutralizing activity is quite difficult. We used cytometric sorting to produce a pool of plasmablasts. We chose the strategy of sorting the entire population of plasmablasts (regardless of their specificity), but blood sampling was performed on day 7 after vaccination of the donor with the Sputnik Light vaccine. Several studies have reported that the number of specific plasmablasts increases in the blood on day 7 or 8 after vaccination or infection [9, 10, 11]. To increase the number of produced hybridomas, we used electrofusion, whose efficiency is an order of magnitude higher than that of fusion with PEG [8]. The use of the K6H6/B5 cell line as partner cells reduced the risk of subsequent segregation of human chromosomes from hybridomas [12]. A total of 12 hybridomas were produced, but only five were RBD-specific, and only one hybridoma (C6D7-RBD) was able to stably synthesize the antibody.

Further analysis revealed that C6D7-RBD belongs to the IgG1 subclass and contains the κ-light chain. C6D7-RBD hMAb is specific to SARS-CoV-2 RBD, with K_D_ = 5.525 × 10^–9^ M.

Traditional techniques used to identify the ability of mAbs to neutralize the SARS-CoV-2 virus are based on assessing the ability of antibodies to inhibit plaque formation or the viral cytopathogenic effect in a sensitive cell culture. Working with the wild-type SARS-CoV-2 virus requires biosecurity and a laboratory environment that meets the safety requirements for working with risk-group II pathogens. Surrogate techniques can be used to facilitate the screening for the neutralizing activity of monoclonal antibodies or sera from recovered/vaccinated donors. These techniques include competitive ELISA that analyzes the effect of antibodies on the interaction between recombinant RBD proteins and ACE-2. This technique reflects the ability of antibodies to inhibit virus entry into the cell. A number of studies have shown that most virus-neutralizing antibodies are specific to the SARS-CoV-2 S glycoprotein RBD, and that their mechanism of action is related to the inhibition of virus binding to the viral receptor ACE-2 on the target cell surface. This technique was used to show that 10-µg/mL C6D7-RBD hMAb almost completely blocks the RBD–ACE-2 interaction.

In world practice, there is experience in the use of hMAb-based drugs. To date, the FDA has only approved three such drugs for emergency treatment of COVID-19. In 2021, the British pharmaceutical company GlaxoSmithKline (GSK) and its American partner Vir Biotechnology introduced sotrovimab (VIR-7831) to the market [13]. The drug comprises a single hMAb specific to the SARS-CoV-2 S protein RBD [14]. In the same year, AbCellera, together with the U.S. Vaccine Research Center at the National Institute of Allergy and Infectious Diseases (NIAID), developed LY-CoV555/bamlanivimab and LY-CoV016/etesevimab [15]. This drug consists of a cocktail of hMAbs specific to the RBD S protein and ACE-2 [16]. The third approved drug, Casirivimab and Imdevimab (REGN10933 and REGN10987), developed by the American biotechnology company Regeneron Pharmaceuticals, consists of two hMAbs specific to the SARS-CoV-2 S protein RBD [17, 18]. Clinical trials have demonstrated that a cocktail of SARS-CoV-2 S protein RBD specific monoclonal antibodies more effectively neutralizes the SARS-CoV-2 virus.

Therefore, the developed C6D7-RBD hMAb is promising and needs further study of its efficacy as a separate hMAb or a component of a mAb cocktail for neutralizing the SARS-CoV-2 virus.

## CONCLUSION

Using cytometric sorting and hybridoma technology, we were able to generate the C6D7-RBD hMAb-producing hybridoma. The C6D7-RBD hMAb belongs to the IgG subclass 1; the light chain is represented by the κ-isotype. The C6D7-RBD hMAb is specific to the SARS-CoV-2 virus in the ability to neutralize its entry into the cell.

## Abbreviations

COVID-19: (COronaVIrus Disease 2019)
SARS-CoV-2: severe acute respiratory syndrome-related coronavirus 2
WHO: World Health Organization
FDA: Food and Drug Administration
hMAb: human monoclonal antibody
mAb: monoclonal antibody
ACE-2: angiotensin-converting enzyme 2
TMPRSS2: transmembrane serine protease 2
RBD: receptor-binding domain
PBS: phosphate-buffered saline
FBS: fetal bovine serum
ELISA: enzyme-linked immunosorbent assay
TMB: 3,3’,5,5’-tetramethylbenzidine
PBS-TW: phosphate-buffered saline containing Tween-20
PAG: polyacrylamide gel
